# Parkinson’s disease as a system-level disorder

**DOI:** 10.1038/npjparkd.2016.25

**Published:** 2016-12-01

**Authors:** Daniele Caligiore, Rick C Helmich, Mark Hallett, Ahmed A Moustafa, Lars Timmermann, Ivan Toni, Gianluca Baldassarre

**Affiliations:** 1 Laboratory of Computational Embodied Neuroscience (LOCEN), Istituto di Scienze e Tecnologie della Cognizione, Consiglio Nazionale delle Ricerche (ISTC-CNR), Roma, Italy; 2 Radboud University Medical Centre, Donders Institute for Brain, Cognition and Behaviour, Department of Neurology, Nijmegen, The Netherlands; 3 National Institute of Neurological Disorders and Stroke (NINDS), Medical Neurology Branch, Bethesda, MD, USA; 4 Western Sydney University, Milperra, NSW, Australia; 5 University of Cologne, Cologne, Germany; 6 Donders Institute for Brain, Cognition and Behaviour, Nijmegen, The Netherlands

## Abstract

Traditionally, the basal ganglia have been considered the main brain region implicated in Parkinson’s disease. This single area perspective gives a restricted clinical picture and limits therapeutic approaches because it ignores the influence of altered interactions between the basal ganglia and other cerebral components on Parkinsonian symptoms. In particular, the basal ganglia work closely in concert with cortex and cerebellum to support motor and cognitive functions. This article proposes a theoretical framework for understanding Parkinson’s disease as caused by the dysfunction of the entire basal ganglia–cortex–cerebellum system rather than by the basal ganglia in isolation. In particular, building on recent evidence, we propose that the three key symptoms of tremor, freezing, and impairments in action sequencing may be explained by considering partially overlapping neural circuits including basal ganglia, cortical and cerebellar areas. Studying the involvement of this system in Parkinson’s disease is a crucial step for devising innovative therapeutic approaches targeting it rather than only the basal ganglia. Possible future therapies based on this different view of the disease are discussed.

## Introduction

Parkinson’s disease (PD) is a neurodegenerative disorder characterized by motor dysfunctions including, among others, tremor, difficulty in initiating and executing voluntary movements (akinesia/freezing/bradykinesia), muscular rigidity, impaired execution of movement sequences as well as by non-motor deficits such as behavioral and cognitive impairments^1–3^ (Appendix Table 1 presents the abbreviations used in the article). The motor dysfunctions of PD are thought to result primarily from the death of dopamine-producing cells in the substantia nigra pars compacta (SNc), an area in the midbrain mainly targeting the striatum (Str), which is the input gate of basal ganglia (BG). As a consequence, PD is characterized by a consistent reduction of striatal dopamine levels.

Common theoretical and empirical approaches studying PD directly focus on such BG alterations.^4^ This view has two important limitations. First, it limits the understanding of the disease as it overlooks anatomical and functional interactions between these nuclei and cerebral cortex/cerebellum. Indeed, BG closely work with cortex (Ctx) and cerebellum (Cer) to form fundamental circuitry involved in motor and cognitive tasks of various complexity, from sensorimotor mapping to reasoning.^5–8^ There is strong evidence demonstrating that Cer and BG receive input from, and send output to, Ctx through multisynaptic anatomically segregated loops performing distinct functional operations.^9,10^ Moreover, recent evidence highlights the existence of an anatomical substrate for a bidirectional communication between Cer and BG. In this respect, studies on rats^11^ and monkeys^12^ have demonstrated that Cer has a strong disynaptic projection to Str via the thalamus (Thal). Recent investigations on cebus monkeys have also shown that the subthalamic nucleus (STN) has a disynaptic projection to the cerebellar cortex via the pontine nuclei (PN).^13^ Similar data have recently been found in humans, using diffusion tensor imaging (DTI).^14,15^ These data have stimulated new research directed at investigating the role of Cer and BG in functions typically associated with cortical areas (e.g., action understanding^7,16,17^), and the role of cerebellar and cortical areas in impairments typically associated with BG, such as Tourette’s syndrome,^18^ dystonia,^19^ and PD.^8,20–23^

Second, focusing on the BG to study PD limits the approaches used for therapy as it implicitly suggests acting on such nuclei in isolation. The most common treatment thus targets the BG and consists of replenishing dopamine depletion. This approach has several drawbacks and might produce a variable response for some motor dysfunctions (e.g., tremor; see refs 20,21,24 for recent reviews). In addition, dopaminergic stimulation can lead to dyskinesia,^24^ impulse control disorders (ICDs),^25^ and may not ameliorate or may even worsen non-motor symptoms.^3,26–28^

**Figure 1:**
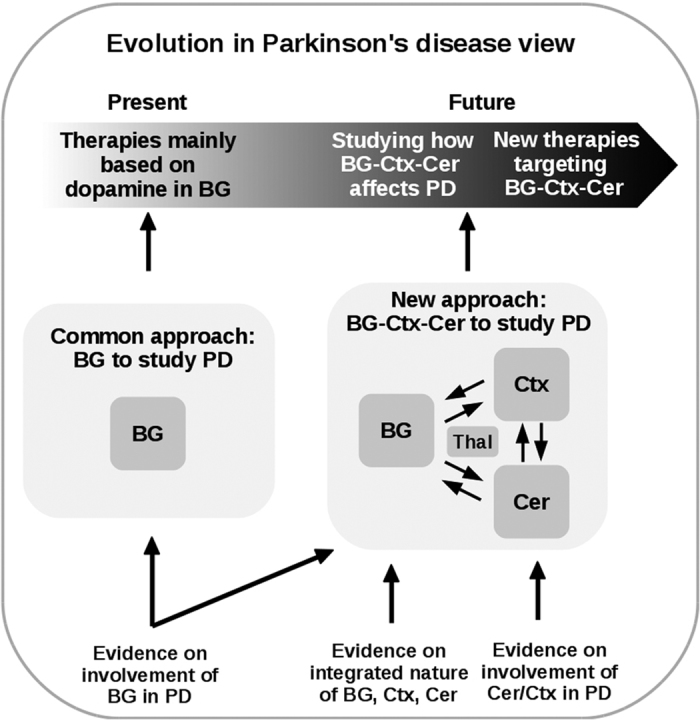
Graphical summary of the systems-level view to the study of PD proposed in this article. Pivoting on evidence supporting the integrated nature of BG, Ctx, and Cer, largely interacting through Thal, and the involvement of Cer and Ctx in PD, the view urges studying PD by focusing on the BG–Ctx–Cer system rather than on BG in isolation. Studying how such system affects PD is a crucial step to draw a more articulated clinical picture of the disease and to devise innovative therapeutic approaches. BG, basal ganglia; Cer, cerebellum; Ctx, cortex; PD, Parkinson’s disease.

This article proposes that investigating the abnormal interactions of the BG–Ctx–Cer integrated system can lead to a better understanding of PD symptoms and treatments. Figure 1 summarizes the system-level view to the study of PD proposed here. This view overcomes the limitations of the single area perspective on PD by supplying a more articulated clinical picture of the disease, and by paving the way to the study of new therapies targeting Cer and Ctx alongside the BG.

More in details, we propose that key PD symptoms (tremor, freezing, action sequencing impairments) can be related to dysfunctions of specific, partially overlapping circuits within the BG*–*Ctx–Cer system. In particular, we provide support for three hypotheses: (i) tremor depends on the abnormal interaction between BG and the Cer–Ctx loops: this hypothesis builds on key data on the involvement of these loops in tremor^20,21,29^ and on the recently discovered STN–Cer disynaptic connection;^13^ (ii) freezing is linked to abnormal interactions between the BG pathways, the presupplementary motor area (pre-SMA) and Cer–Str disynaptic link:^12^ this hypothesis builds on evidence showing the influence of pre-SMA^30,31^ and Cer^31,32^ on freezing; (iii) dysfunctions in the circuit linking Cer to SMA, and in the hyperdirect pathway linking SMA with STN, may have a role in action sequencing impairments: this hypothesis is based on data showing the contribution of pre-SMA/SMA,^33,34^ Cer^17,35^ and BG^36,37^ in action sequences and on recent evidence on pre-SMA/SMA role in PD.^38,39^ We also furnish some more general indications on how the view proposed here might contribute to advance our understanding of other motor and non-motor features of PD. The complexity of the cortical and subcortical circuits underlying PD deficits suggests using dynamical multi-scale computational models to capture its quantitative aspects and to integrate data from multiple sources, such as different brain imaging techniques. This opens up the possibility to design new procedures for monitoring and treating the disease, that focus on the whole BG–Ctx–Cer system rather than BG in isolation.

## The neural system underlying PD

**Figure 2:**
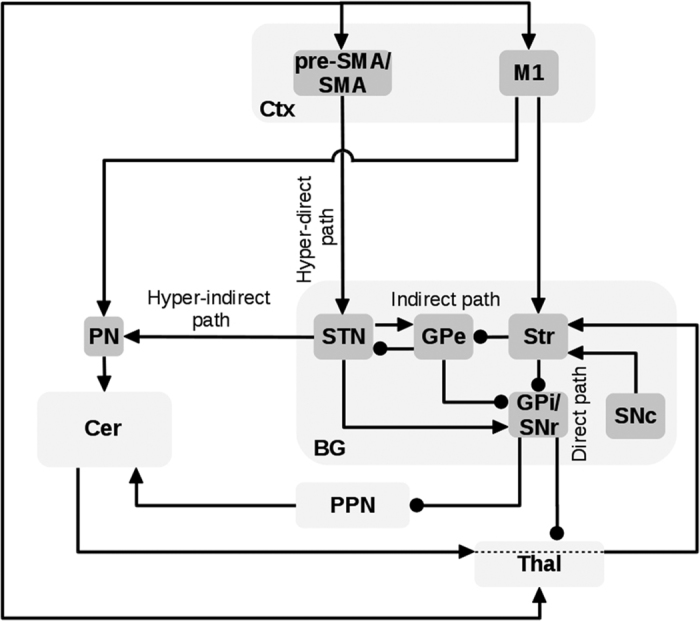
Schema of the basal ganglia-cortical-cerebellar (BG–Ctx–Cer) system involved in three PD motor symptoms, in particular tremor, freezing, and action sequence impairments. The arrows indicate glutamatergic excitatory connections whereas lines ending with a filled circle represent inhibitory GABAergic projections. The bidirectional arrows linking Thal and Ctx include both the BG-cortical and the cerebellar-cortical channels (note that Cer sectors within Thal could be partially overlapped with those of BG, see Ref. 141 for more details). The dashed lines within Thal represent the cerebellar target sectors within Thal through which the Cer reaches Str.^12,13^ BG, basal ganglia; Cer, cerebellum; Ctx, cortex; GPe, external globus pallidus; GPi, internal globus pallidus; M1, primary motor cortex; PPN, pedunculopontine nucleus; PN, pontine nuclei; pre-SMA, pre-supplementary motor area; Str, striatum; STN, subthalamic nucleus; SNr, substantia nigra pars reticulata; SNc, substantia nigra pars compacta; SMA, supplementary motor area; Thal, thalamus.

**Figure 3:**
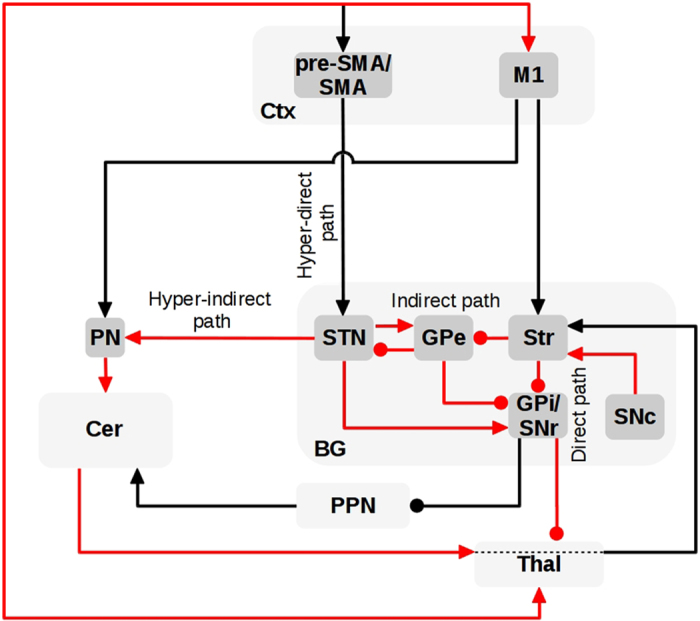
Cortical–subcortical circuit underlying Parkinson’s disease (PD) tremor. The red arrows indicate the anatomical pathways through which the elements of the cortical–subcortical system may interact to produce tremor in PD.

**Figure 4:**
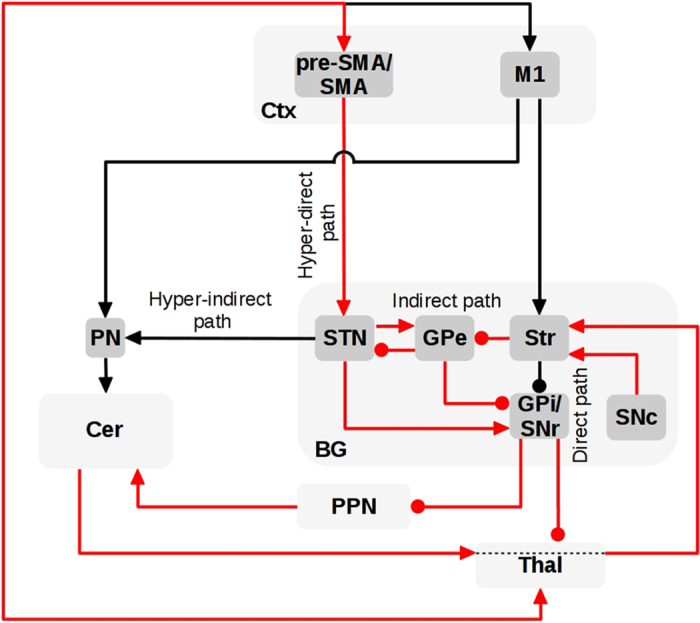
Cortical–subcortical circuit possibly underlying Parkinson’s disease (PD) freezing. The red arrows indicate the anatomical pathways through which the elements of the cortical–subcortical system interact between them to produce freezing.

**Figure 5:**
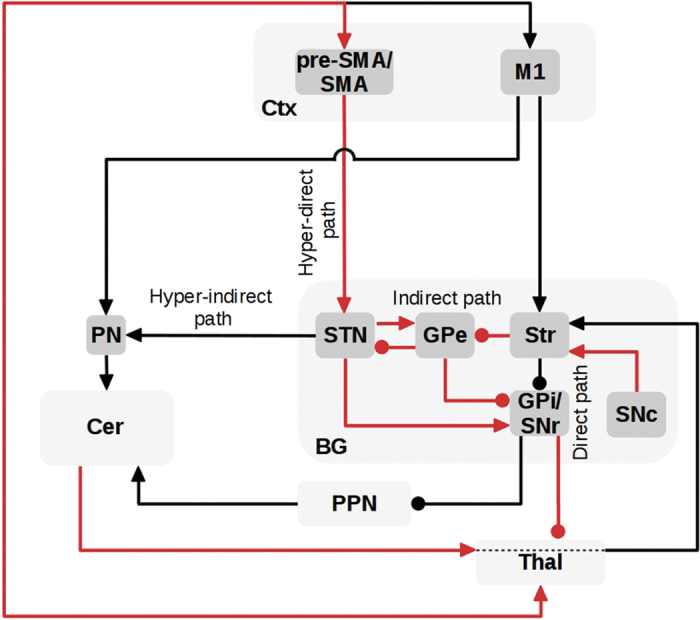
Cortical–subcortical circuit possibly underlying Parkinson’s disease (PD) action sequencing impairments. The red arrows indicate the anatomical pathways through which the elements of the cortical–subcortical system interact between them to produce action sequencing impairments.

To illustrate with specific cases the utility of the proposed perspective to better understand proximal causes of PD, in this section we explain how three partially overlapping cortical–subcortical circuits may underlie three important PD symptoms. Figure 2 shows some key components of the BG–Ctx–Cer system that are important to study the three symptoms. The schema is not exhaustive of all the possible connections between basal ganglia, cortical, and cerebellar areas. Rather, it focuses on the connections that may have a major role in the three PD symptoms considered here. This is the reason why, for example, the figure indicates SMA/pre-SMA as the only sources of the hyperdirect pathway from cortex to STN, omitting the projections from M1 to STN. The same considerations hold for the Figures 3, 4, 5, which are derived from Figure 2. The other pathways not considered here might have roles in other aspects of PD symptoms.

### The cerebral network underlying Parkinson’s tremor

The occurrence of resting tremor in PD is probably related to the death of SNc dopamine-containing cells. PD tremor mainly involves dysfunctions in the system formed by the motor cortex, cerebellum, thalamus, and basal ganglia.^40–42^ Patients with tremor-dominant PD show an increased functional connectivity between BG and the cerebello-thalamo-cortical circuit.^29^ This evidence suggests that PD tremor may result from a pathological interaction between BG and the cerebello-thalamo-cortical circuit (Cer–Thal–M1). However, the specific mechanisms underlying such pathological interaction are still widely debated.^43^

Several hypotheses have been proposed to explain the occurrence of tremor in PD (see ref. 20 for an overview). Most of these have largely focused on locating the tremor pacemaker in thalamus^44^ or BG,^45^ or in pallido-thalamic interactions.^46^ As underlined in ref. 20, these hypotheses are unable to explain the involvement of both cerebello-thalamo-cortical system and BG in PD resting tremor.^40,41^ To address this issue, Helmich *et al.* have recently proposed a system-level explanation of PD resting tremor called the “dimmer-switch” hypothesis.^20,29^ According to this hypothesis, BG work analogously to a light switch that triggers tremor-related responses in the Cer–Thal–M1 circuit and this, in turn, generates tremor modulating its intensity like a light dimmer. This hypothesis is in line with previous theoretical proposals highlighting the role of BG for movement initiation and of Cer for movement amplification,^47^ and represents an important attempt to account for tremor in PD through a system-level perspective. However, this hypothesis does not specify how BG could interact with the Cer–Thal–M1 circuit to switch on tremor. Here we extend the dimmer-switch model by highlighting possible pathways through which BG could interact with the Cer–Thal–M1 circuit (Figure 3).

We hypothesize that the main axis involved in producing tremor is the Thal–Ctx system including both the BG–Thal–M1 and the Cer–Thal–M1 circuits. This hypothesis is based on previous studies showing that thalamo-cortical functional connectivity distinguishes pathological tremor from mimicked tremor,^48^ that dopamine specifically reduces thalamo-cortical coherence in PD tremor,^49^ and that Thal has strong rhythmic properties capable of driving the tremor on a cycle-by-cycle basis.^50^ The BG are connected with the thalamo-cortical system via the direct and indirect pathways that exert a net inhibitory influence onto Thal through GPi. Thal, in turn, is bi-directionally connected with M1 through excitatory links. In non-pathological conditions, the inhibition of specific output nuclei of BG leads to increased activity within the circuits linking Thal with M1 allowing the focused facilitation of specific motor patterns.^51^ Through these pathways, BG–Thal–M1 and Cer–Thal–M1 circuit converge at the level of M1.^7,10^ Specifically, GPi sends GABAergic projections to the anterior part of the ventrolateral Thal which in turn sends excitatory efferents to the M1. M1 projects to the posterior part of the ventrolateral Thal that also receives cerebellar projections.^52^ This hypothesis would explain why deep brain stimulation (DBS) of STN, GPi, and Thal ventral intermediate nucleus (VIM, which is a target of cerebellar efferents) are all effective in treating tremor.^53–55^

Another hypothesis is that the recently discovered STN-cerebellar cortex anatomical link through the pontine nuclei^13^ may have an important role in PD tremor by influencing the dysfunctional interaction between BG and the thalamo-cortical system. In particular, we propose that the subthalamic-pons-cerebellar circuit (STN–PN–Cer)^13^ may be considered as a further pathway, alongside the traditional ones (direct, indirect, and hyperdirect pathways), which allows the BG to influence the excitability of the thalamo-cortical system through Cer. We term this the “hyper-indirect pathway”. The projections from STN to PN, and from PN to granule cells of cerebellar cortex, are long-range and therefore probably glutamatergic.^13^ Increased STN activity, as found in PD,^56^ would thus excite the cerebellar cortex, which in turn has an inhibitory influence onto the deep cerebellar nuclei.^57^ Further investigations are needed to confirm this hypothesis as there is no direct evidence supporting the impact of STN descending glutamatergic projections on PN and cerebellar neurons. As the deep cerebellar nuclei have glutamatergic projections to the VIM (cerebellar thalamus; refs 58,59), the net result would be inhibition of the thalamo-motor system. *In vitro* studies have shown that hyperpolarization of single thalamic neurons turns these neurons into single-cell oscillators with a firing frequency of about 6 Hz, which is the resting tremor frequency in PD.^50^ In PD, changes in the influence of the direct/indirect pathways, the “hyper-indirect” pathway, or the balance between them, onto the thalamo-motor system, may be responsible for triggering rhythmic activity in the Cer–Thal–M1 circuit. A recent dynamic causal modeling functional MRI (fMRI) study in tremor-dominant PD tested how tremor-related activity is transmitted from the basal ganglia toward the cerebello-thalamo-cortical circuit, comparing the contribution of the two pathways outlined above. The findings suggest that tremulous activity first arises in the GPi, and is then propagated through effective connectivity from the GPi toward the motor cortex (rather than the hyper-indirect pathway from STN to Cer^60^). After the onset of tremor, tremor-related peripheral influences onto Cer may further modulate processing within the Cer–Thal–M1 circuit, having a role in maintaining the tremor once it has been triggered by the BG.^61^

Support for our cortical–subcortical circuit hypothesis underlying tremor comes also from analysis of the tremor-related activity in the areas considered in Figure 3 (refs 21,62). Overall, on the methodological side this analysis shows how a system-level perspective is needed to disentangle the complex involvement of different neural circuits in the production of PD tremor.

### Freezing as response conflict impairment: cortical–subcortical substrates

Freezing is the inability to begin or continue a voluntary discrete or rhythmic movement. It can affect walking, writing, speech, and is also associated with deficits in a number of executive functions including attention and conflict resolution.^63–66^ The vast range of conditions provoking or relieving freezing supports the involvement of a complex brain network including both cortical and subcortical areas.^65,67^

Pivoting on the anatomical connections between BG and Ctx^8,10^ and between BG and Cer,^13,14^, and on recent data about the involvement of pre-SMA^30,31,63,68^ and Cer^31,32,63^ in freezing, we propose here some system-level hypotheses on the possible alterations of cortical–subcortical circuits that might underlie freezing (Figure 4). Striatum modulates the output nuclei of BG through two pathways. The first is the direct pathway that involves a Str GABAergic connection directly inhibiting GPi and substantia nigra pars reticulata (SNr). The second is the indirect pathway involving two sub-routes: the short indirect pathway, linking the external globus pallidus (GPe) to GPi/SNr via GABAergic connections; and the long indirect pathway linking GPe to STN which in turn projects to GPi/SNr.^69^

Low dopamine levels in PD lead to a reduction in the strength of striatal inhibition to GPi/SNr and an increase in striatal inhibition of GPe. A lower activation of GPe, in turn, results in an increased GPi/SNr inhibitory output both via the Str–GPe–GPi/SNr short indirect pathway and via the Str–GPe–STN–GPi/SNr long indirect pathway.^2^ The GPi/SNr abnormal inhibition of BG target areas is relevant for the manifestation of multiple PD symptoms including freezing.^2,30^ In particular, this inhibition affects Thal–M1 circuits subserving the performance of discrete voluntary movements.^70^ Moreover, this inhibition might abnormally inhibit pre-SMA and SMA, namely cortical areas that have a critical role in the initiation, sequencing and termination of movements.^33,34,38^ These areas have been often found to be underactivated in PD patients,^30,31^ putatively because as other thalamo-cortical areas they receive an abnormal inhibition from BG,^22,23^ or due to other internal disregulations.^30,68^ In addition, an increase of inhibitory output onto the dorsal pedunculopontine nucleus (PPN) and Thal might contribute to freezing of rhythmic motor behaviors,^32,71^ such as walking, due to the PPN efferent connectivity to the central pattern generators of the spinal cord coordinating the alternating activity of flexor/extensor muscles.^72–75^ The involvement of PPN in freezing is indirectly supported by recent data showing that PPN DBS may reduce freezing of gait in PD patients.^76^ Further, PPN receives inputs from STN, GPi and SNr^77^ and projects into the STN, SNc, GPi, Cer, spinal cord, and SMA.^77,78^ In general, PPN can have a role in motor modulation and in the initiation and maintenance of locomotion.^79^ In PD patients it has been shown that PPN activity changes during movement preparation and execution.^80^ Building on recent data,^81^ we propose that PPN may abnormally regulate STN activity through the Cer–Thal–Str pathway, thus further augmenting freezing. Indeed, recent evidence shows that stimulation of PPN may influence Cer activity.^32,78,82^ The influence of PPN on Cer may in turn increase BG output by regulating STN activation through the Cer-Thal-Str newly discovered pathway^6,12^ and the Str–GPe–STN–GPi/SNr long indirect pathway considered above. A reduced cerebellar connectivity with other areas involved in motor control^31^ might also lead to a lack of on-the-fly corrections of posture and gait causing balance problems and walking abnormalities.^83^ A reduced coordination of steps during challenging gait phases, such as turning, may precipitate freezing.^63^

### Action sequencing impairment as deficit in timing: cortical–subcortical substrates

PD patients have a difficulty in performing action sequences, including completing sequences of heterogeneous movements in correct order.^84,85^ Here we discuss a cortical–subcortical circuit that may underlie this symptom. The circuit, shown on Figure 5, is mainly based on data supporting the contribution of pre-SMA, SMA, Cer and BG in managing action sequences.^17,33,36,37^ Different types of neurons in pre-SMA and SMA help to encode not only where in a sequence the action is but also the conditional links between the previous response and the upcoming response, often in a highly specific manner.^38^ In this respect, it has been shown that pre-SMA and SMA neurons respond before some sequences (e.g., turn-pull-push a lever) but not others (e.g., turn-push-pull),^34^ that some neurons of pre-SMA and SMA respond only to the rank order of a movement in the sequence (e.g., only before the second movement regardless of what the movement is),^86^ and that pre-SMA and SMA cells also encode the number of movements that remain to be made to complete a sequence to obtain a reward.^87^

As both pre-SMA and SMA are anatomically linked to Cer through Thal,^88^ Cer could assist pre-SMA and SMA by contributing to the anticipatory activation of the neurons of these cortical regions during action sequences. In particular, pre-SMA and SMA, working in synergy with Cer, may possess the capacity of anticipating future events at fast temporal scales based on forward models.^7,35^ This view is in line with the “timing hypothesis” of cerebellar function postulating that Cer is critical for representing the temporal relationship between task-relevant events as it works as a general “timing co-processor” whose effect depends on the targeted centres.^89^ The circuits linking BG with pre-SMA and SMA may also support the processes underlying action sequencing. In more detail, the activation of pre-SMA and SMA neurons could regulate, in an anticipatory fashion with respect to the next movement, the activation of STN though the hyperdirect pathway. This pathway, in turn, conveys the signal from motor-related cortical areas (in this case pre-SMA and SMA) to the globus pallidus, bypassing the striatum, with shorter conduction time than the signal conveyed through the striatum.^90^ The anticipatory activation of STN could indirectly support the anticipatory activation of the next movement by fostering the movement selection processes through the direct pathway of BG.^2,51^

These neural processes might be dysregulated in PD. In particular, in the circuit shown in Figure 5 the triggering event leading to an action sequencing impairment may be related to the malfunctioning of the BG-preSMA/SMA circuit, caused by dopamine dysregulation, and this might propagate to the Cer–Thal–pre-SMA/SMA circuits, which support the timing aspects of pre-SMA/SMA functioning.^34,38,86^ As a consequence, pre-SMA and SMA neurons may not regulate as needed the activation of STN through the hyperdirect pathway. In turn, the lack of anticipatory activation of STN may fail to support the activation of the next movement, thus producing impaired action sequencing.

### A system-level view of other PD symptoms

A system-level perspective might also help to disentangle unclear aspects related to other PD symptoms, although for these a formulation of hypotheses as articulated as those proposed for tremor, freezing and action sequences requires further investigations. Akinesia, for example, has been linked to the increased inhibitory BG output. Indeed, DBS applied to the globus pallidus has been shown to reduce akinesia symptoms in Parkinsonian monkey models.^21,91^ It has also been shown that beta band activity in the STN correlates with akinesia in PD, and that the clinical improvements following DBS of STN can affect both akinesia and rigidity.^92^ Further, transcranial stimulation reduces rigidity in PD patients suggesting a role for the cortico-spinal pathway.^21,92^ It has also been found that the Cer is overactive in PD patients with akinesia in comparison with healthy controls, suggesting that the Cer may have a compensatory role.^21^ However, it is not clear through which neural pathways Cer can enhance movement speed and reduce akinesia although one might expect this to involve the Cer–Ctx loops and the aforementioned newly discovered Cer–Thal–BG pathway.^12^ It is also unclear how to integrate the role of the Cer as a compensatory system and as a motor timing system,^21^ but it is expected that an explanation has to involve system-level mechanisms.

In the same line, a system-level perspective involving cortical–subcortical brain regions could be used to characterize non-motor deficits in PD. One example is ICDs, which include pathological gambling, compulsive eating, and hypersexuality.^93^ ICDs might be caused by an overstimulation of D2 receptors.^25^ Impulsivity can also be caused by STN DBS.^94^ Like motor symptoms discussed above, ICDs in PD are also associated with cortical and subcortical dysfunctions.^95^ Besides the indirect pathway including STN, other studies found that the nucleus accumbens also has a role in the occurrence of ICDs.^96^ Other neural studies have implicated other brain regions including the hippocampus,^97^ prefrontal cortex,^98^ and amygdala.^95^ ICDs in PD have been shown to be related to PD motor impairment, for example, patients with gait impairment show more impulsive behavior than tremor-dominant patients.^99^ One study also found that motor complications are more common in PD patients with ICDs than in patients without ICDs.^100^

Inducing anxiety was found to exacerbate freezing of gait in PD patients.^101^ Further, prefrontal-based processes, including attentional control, executive function, and working memory, were shown to impact successful upper and lower limb motor control.^102,103^ Clinical and functional imaging studies suggest that cognitive symptoms in PD might be linked to the involvement of prefrontal, premotor and parietal regions,^23,27,39^ hippocampus,^26,104^ and amygdala.^28^ In addition, increasing evidence suggests that the cognitive decline reported in PD patients is not simply related to a mere dopaminergic deficit.^105^ Indeed, neurodegeneration in PD, besides affecting SNc dopaminergic neurons, also appears in serotonergic neurons in the raphe nuclei,^106^ noradrenergic neurons in the locus coeruleus,^107^ and in cholinergic neurons in the basal forebrain complex.^108^ An enhanced neural architecture accounting for system-level balancing of dopamine, noradrenaline, serotonin, and acetylcholine could help understand the roles played by these neuromodulators in PD non-motor deficits. Overall, this evidence suggests that extending the system-level schema proposed here to include other key cortical and subcortical areas relevant for motor and cognitive control^109,110^ could be useful for understanding non-motor deficits associated with PD and their relationship with motor dysfunctions.^4,111–113^

### From theory to computational models of the BG–Ctx–Cer system imparements in PD

The brain system proposed in Figure 2 as the cortical–subcortical network producing PD symptoms has a clear, distinct feature: it involves highly recurrent circuits. In this article, we aimed to link PD symptoms to specific impairments of the network, but this exercise has an inherently limited scope: it can only partially disentangle the circular causations involved by the above-discussed circuits, and on this basis offer quantitative predictions. For example, we proposed specific hypotheses to explain tremor and freezing, but it is not possible to establish if such hypotheses are self-consistent and sound only on a verbal basis. Computational models have the power to prove the self-consistency of hypothesis as those proposed here.^4,51,69,94,109,114^ This is a necessary condition to establish the validity of theories (although it is not sufficient and has to be followed by empirical examinations). For this reason, we consider here possible approaches to follow to translate the verbal theories presented here into operational computational models able to offer sound explanations and quantitative predictions on PD symptoms.

The models used to this purpose should have two key ingredients. First, to be able to capture the local and global dynamic functioning of the brain neural systems involved in PD they should be multi-scale models.^109,115^ Models in computational neuroscience often deal with a single-scale description that corresponds to a particular anatomical scale; for example, the cellular scale, the microcircuit scale, the meso-level (e.g., the relations between BG nuclei), and the system-level (e.g., the BG–Thal–Ctx–Cer system). In particular, a deeper understanding of PD will come only from analyses and models spanning multiple spatial and temporal scales. Indeed, striatal dopamine loss can be described at the cellular level, but PD symptoms can be understood only by tracing the effects of such loss onto the micro-/meso-circuits of BG and how such effects reverberate onto the BG–Ctx–Cer system. To this purpose, computational model components with high anatomical detail could be embedded within more abstract cortical–subcortical model components.^115^ For example, a detailed model formed by conductance-based neurons might be integrated into the striatal microcircuit. This, in turn, could be embedded in a spiking neuron model of the BG–Ctx–Cer. In this way, phenomena happening at the lowest level of the embedded hierarchy—where the dysfunctional pathology of PD originates—may cascade upwards to affect upper levels of the hierarchy.^116^

Second, computational models of the PD cortical–subcortical network should capture the dynamical events characterized by circular causality involving the cortical–subcortical network. In this respect, it has been proposed that the fundamental information processing happening within (and between) BG, Ctx and Cer are highly dynamic and so can be captured only through highly dynamic computational models (e.g., BG: Refs, 114,116; Ctx: Refs, 117,118; Cer: Refs, 89,119). Capturing such dynamic processes with quantitative models will lead to a deep understanding of the interplay of cortical/sub-cortical areas and how they might be altered in PD, thus supporting the development of new monitoring techniques and therapies.

## New approaches for monitoring and treating PD

The system-level view of PD features has a high potential for the development of innovative procedures for monitoring and treating PD. We show this by illustrating some of these possibilities. Regarding monitoring, one of the biggest future challenges for imaging techniques applied to PD is to integrate their results to identify the mechanisms that might be targeted with drugs and other interventions.^3,120,121^

The dynamic multiscale computational models discussed in the previous section could support the integration of the data acquired with different techniques based on their capacity to incorporate information at different levels. At the system- and meso-level, the models could integrate data from resting-state functional magnetic resonance imaging (rsfMRI), structural magnetic resonance imaging (sMRI), arterial spin labeling (ASL) on the BG–Ctx–Cer overall activity. These techniques could be used to measure parameters related to the entire BG–Ctx–Cer network^122^ and this information could be used to constrain the overall architecture of the model. Recent fMRI analysis techniques, such as dynamic causal modeling,^123^ use Bayesian statistics to systematically compare the evidence for different functional network configurations underlying different behaviors.^124^ These methods have been used for example to test the circuit-level influences of STN-DBS in PD,^125^ or the circuit-level changes underlying dyskinesias^126^ and bradykinesia.^127^ Another method is to use functional network connectivity analyses to reveal connectivity patterns among different brain regions.^123^ Using these techniques it has been found that the disconnection between the prefrontal cortex and BG in PD could explain some aspects of freezing of gait.^128^ Techniques returning more detailed information on the actual anatomical connectivity, such as DTI, could be used to further constrain the model architecture. DTI has been extensively used with healthy human subjects to investigate BG–Ctx connectivity patterns^129^ and so it might be used to also furnish data on Cer–Ctx and Cer–BG connections in both healthy and PD subjects. Linked to this, 18F-fluorodeoxyglucose positron emission tomography (PET) has shown changes in Cer and Ctx connectivity patterns in relation to tremor in PD.^130^ DTI could also furnish information on the internal connectivity of areas, for example within the BG.^131^ At the micro-circuit and cellular level, data on dopamine production and receptors from PET and from dopamine transporter-single photon emission computed tomography (DAT-SPECT) could be used to set the model parameters so as to capture specific PD damages.

By disentangling the multifaceted mechanisms underlying PD symptoms, the system view of PD proposed here, operationalized into dynamical system-level models integrating multi-source data, could lead to a systematic data-driven improvement of therapies.^120,132^ We support this possibility by referring to some relevant treatment techniques. DBS and transcranial magnetic stimulation (TMS) have been used with various brain targets, in particular sites involving areas of the BG–Ctx–Cer system and not only BG. Indeed, several experiments support the idea that DBS preferentially modulates remote structures rather than local circuits since fibers of passage are more excitable than local cell bodies at the site of stimulation.^133^ The analysis of the cortical–subcortical circuits discussed above, possibly supported by computational models integrating multiple data sources, could represent a necessary step to better understand the mechanisms underlying DBS/TMS effects and so to identify possible new targets within the BG–Ctx–Cer system.

Regarding tremor, the literature suggests that this symptom can have a variable responsiveness to dopamine treatments.^20,21,24^ Alternative effective therapies for tremor are based on DBS of STN, of the posterior subthalamic area, and of the thalamic ventral intermediate nucleus.^53–55^ The improvements in tremor after DBS of STN could be indirectly related to a modulation of the Cer activation as suggested by several imaging studies reporting metabolic changes in Cer during DBS of STN.^21,134^ In general, it has been shown that the efficacy of DBS for treating tremor may be improved when taking interregional structural or functional connectivity of BG, Cer, Thal, and M1 into account.^58^ The circuit shown in Figure 3 supports this perspective by providing a hypothesis on how the STN-Cer–Thal–M1 and the STN–GPi/SNr–Thal–M1 circuits may interact during tremor. Moreover, the analysis of this circuit suggests that it is important to further investigate how the stimulation of Cer (for example, through TMS)^21^ and M1 (e.g., with rTMS^135^) may affect tremor, and whether functional uncoupling of BG from the Cer–Thal–M1 circuit could reduce tremor.

The lack of fundamental understanding of the neural mechanisms underlying freezing limits current therapeutic options. Freezing is weakly responsive to dopaminergic medication^30^ and also shows a variable response to DBS therapy targeting either GPi, STN or PPN.^136^ In this respect, there are data showing that STN of GPi DBS are not effective in reducing freezing of gait, although preliminary evidence suggests that PPN DBS may reduce it.^76,136^ The circuit shown in Figure 4 suggests that alternative target areas for the treatment of freezing in PD could be the Cer or pre-SMA. This would be supported by various recent studies showing reduction of dyskinesia^137^ and improvements in handwriting^138^ in PD patients after TMS stimulation of SMA circuits. These data may provide the foundation for a larger investigation of the effects of noninvasive brain stimulation over the pre-SMA and SMA in individuals with PD.

Multi-scale and dynamical computational modeling studies can simultaneously investigate potential treatments for tremor, freezing, and action sequencing impairments. This approach could provide a breakthrough in devising new therapies for PD as it could allow testing drug effects (and collateral effects) in simulation (https://ec.europa.eu/digital-agenda/en/virtual-physiological-human and see also the EU research project NoTremor: http://notremor.eu/notremor/) and use them to select the most promising therapeutic interventions that produce the simultaneous reduction of several symptoms while reducing side effects.

This paves the way to the development of even more innovative therapeutic approaches. PD symptoms are highly variable in different patients and we currently lack widely-accepted systematic ways to adapt therapies and medications to them.^1,3^ The adoption of a system-level view of PD features, supported by multi-scale models, could be the necessary step to trace individual brain differences underlying different patients subtypes, for example tremor-dominant versus akynetic-rigid.^20,21^ This knowledge would thus furnish an invaluable basis on which to tailor interventions on the specific features and conditions of the specific patient.

## Conclusions

After Alzheimer’s disease, PD is the most common neurodegenerative disorder worldwide. It primarily affects the elderly and thus due to population aging it has become a rapidly growing area of concern. Owing to the high prevalence of the disease (about 6.3 million people around the world) the limitations of pharmacotherapy and neurosurgery remedies, and the social and economic burden of PD,^24,139,140^ innovative approaches to the study and treatment of PD are needed. We argue here that to strengthen our understanding of the wide spectrum and variability of PD motor symptoms we need to address how dopamine dysregulation reverberates on the whole BG–Ctx–Cer system. This broader perspective allows for understanding the dopamine-related causes of PD symptoms as linked to the circular dynamic relations involving the meso-level multiple circuits within BG, Cer and Ctx and their reciprocal interactions at the level of the whole system. This perspective also supports the identification of new possible disease monitoring processes and therapeutic interventions, for example, the identification of new targets for DBS and TMS, a model-based guidance of brain imaging techniques to follow the disease evolution, and a more informed solution of drug-related side effects concerning psychiatric and cognitive disorders.

## Figures and Tables

**Table 1:** Main abbreviations used in the article

ASL	Arterial spin labeling	PN	Pontine nuclei
BG	Basal ganglia	PPN	Pedunculopontine nucleus
Cer	Cerebellum	pre-SMA	Pre-supplementary motor area
Ctx	Cortex	rsfMRI	resting-state functional magnetic resonance imaging
DAT-SPECT	Dopamine transporter-single photon emission computed tomography	rTMS	Repetitive transcranial magnetic stimulation
DBS	Deep brain stimulation	sMRI	structural magnetic resonance imaging
DTI	Diffusion tensor imaging	SMA	Supplementary motor area
GPe	External globus pallidus	SNc	Substantia nigra pars compacta
GPi	Internal globus pallidus	SNr	Substantia nigra pars reticulata
ICDs	Impulse control disorders	STN	Subthalamic nucleus
M1	Primary motor cortex	Str	Striatum
PET	Positron emission tomography	Thal	Thalamus
PD	Parkinson’s disease	TMS	Transcranial magnetic stimulation
PFC	Prefrontal cortex	VIM	Ventral intermediate nucleus of thalamus

## Appendix

Table 1
